# Gas fermentation: cellular engineering possibilities and scale up

**DOI:** 10.1186/s12934-017-0676-y

**Published:** 2017-04-12

**Authors:** Björn D. Heijstra, Ching Leang, Alex Juminaga

**Affiliations:** LanzaTech, Inc., 8045 Lamon Ave, Suite 400, Skokie, IL USA

**Keywords:** Climate change, GHG, Waste gas, Syngas, Fermentation, Gas contaminants, Carbon recycling, Carbon capture and utilization, Scale up

## Abstract

Low carbon fuels and chemicals can be sourced from renewable materials such as biomass or from industrial and municipal waste streams. Gasification of these materials allows all of the carbon to become available for product generation, a clear advantage over partial biomass conversion into fermentable sugars. Gasification results into a synthesis stream (syngas) containing carbon monoxide (CO), carbon dioxide (CO_2_), hydrogen (H_2_) and nitrogen (N_2_). Autotrophy–the ability to fix carbon such as CO_2_ is present in all domains of life but photosynthesis alone is not keeping up with anthropogenic CO_2_ output. One strategy is to curtail the gaseous atmospheric release by developing waste and syngas conversion technologies. Historically microorganisms have contributed to major, albeit slow, atmospheric composition changes. The current status and future potential of anaerobic gas-fermenting bacteria with special focus on acetogens are the focus of this review.

## Background

The critical need for technologies to limit greenhouse gas (GHG) outputs and slow down warming of the Earth is rapidly accepted. Essential to this is a further improvement in global awareness of nations and generations, and their demand for sustainable technology development and products. At the same time continued questioning of polluting industry and government enforced further tightening of emission rules is essential. Sadly, 2016 marks the year the global atmospheric CO_2_ level, measured at Mauna Loa Observatory, permanently reached values over 400 ppm [1]. This level is thought to have an impact extending far beyond our lifetime and the link to the increasing average global temperature is undeniable. On the 14th of November 2016 the World Meteorological Organization (WMO) reported at the 22nd session of the Conference of the Parties (COP22) United Nations global climate summit in Morocco that 2016 is on track to be the hottest year on record. The vast amount of published research on climate change is unanimous and unequivocal pointing to the carbon footprint of the expanding world population. The urgency to reduce emissions and divest from fossil fuels has been recognized by World leaders from over 190 countries who negotiated the Paris Agreement at the 21^st^ Conference of the Parties to the United Nations Framework Convention on climate change [2]. This agreement was signed by 174 countries on 22 April 2016 in New York and each country that ratifies the agreement will have to set emission reduction or limitation targets, known as “nationally determined contribution,” or “NDC,” however the targets will be voluntary [2].

## Available gaseous feedstocks

A variety of large scale industrial processes generate side streams containing low to medium and high BTU (British Thermal Units) value off gases. Examples are steel mills, ferroalloy industries, refineries and chemical plants producing high CO containing gases with variable compositions of H_2_, CO_2_, CH_4_ and N_2_. Many of these gases are flared or preferably burned for internal energy generation within the production facility. Another large gas source, biomass gasification to generate fermentable syngas, is recognized as an alternative to lignocellulosic biomass to fuel conversion. Virtually any waste product can be recycled by turning this into syngas [3–6].

When derived from biomass, syngas can be variable in H_2_ (1.2–7.3 mol%) [7, 8] which makes this less suited for catalytic processes such as the Fischer–Tropsch Process (FTP) which require a fixed H_2_:CO ratio of 2:1 [9, 10]. In addition, non-lignocellulosic biomass gasification such as municipal solid waste (MSW) is another rapidly growing gas source with limited impact on land usage and a preferred technology in crowded nations. Within petrochemical refineries (syn)gas or natural gas to liquid (GTL) technologies are well developed but require high capital investment to be economically viable and compared to petroleum based fuels have high greenhouse gas (GHG) output [11]. Within petrochemical refineries several streams of ‘stranded gas’ often remain underutilized due to logistical and economic barriers [12]. To limit carbon emissions into the atmosphere governments are increasingly exploring regulatory incentives while planned CO_2_ capping can provide economic benefits [13]. New regulatory opportunities can be expected to arise, further growing the gaseous pool available for conversion by gas fermentation.

According to life cycle analysis (LCA) studies, in many of the feed stock examples mentioned above a microorganism based gas to liquid conversion could be an economically profitable proposition while simultaneously decreasing GHG emissions when compared to fossil gasoline [14, 15].

## Gas fermentation process

The advantages of gas fermentation have been made clear in recent reviews [16–19]. The available macro gas composition determines the organisms available for conversion: autotrophic acetogenic, carboxydotrophic, and methanotrophic bacteria can fix the carbon from CO, CO_2_ or CH_4_ containing gases, respectively. Although chemical processes are generally faster than biological conversions, the high enzymatic specificities of biological reactions result in higher product selectivity with the formation of fewer by-products.

In this review we present data from acetogens which can conserve energy through CO_2_ (CO) fixation via the Wood-Ljungdahl pathway (WLP). This is the most efficient known pathway to convert CO_2_ to secreted organic products [20, 21]. The key intermediate of the WLP, acetyl-CoA, is a precursor for enzymatic production of various other organic compounds, production of which can be of commercial interest [20, 22–25].

H_2_ can provide an additional energy source and certain acetogens are able to grow and produce ethanol from CO_2_ and H_2_ [26], providing direct CO_2_ sequestration into products. Direct input of wind, hydro or solar generated electrons could further improve carbon capture utilization (CCU) in these naturally occurring microbial cell factories. Sakimoto *et all* showed a remarkable biomimetic approach with direct electron input into the WLP of *Moorella thermoacetica* by photosensitizing these nonphotosynthetic microbes using a biological-inorganic hybrid approach. This is a true solar to chemical carbon dioxide reduction with 90% selectivity to acetate and 10% selectivity to biomass [27]. A wide variety of CO_2_ reduction technologies remain under development and each could have its own positive impact reducing atmospheric CO_2_ levels [28–31].

A critical aspect of any fermentation involving gases as a substrate is the ability of the gas to solubilize in the liquid to a concentration that does not inhibit microbial metabolism. Inhibition can occur by the substrate being too concentrated [32] or by a low volumetric mass transfer coefficient (k_La_) when substrate availability can become rate-limiting. A variety of reactor configurations attempting to achieve an optimal and controllable k_La_ have been extensively discussed in the literature: Continuous stirred tank reactors (CSTR’s), bubble columns, loop reactors, immobilized beds, and hollow fiber membrane columns each have certain process dependent benefits and specific volumetric mass-transfer coefficients [4, 6, 18, 33–36].

## Detailed gas composition

The wide variety of industries producing waste gas streams invariably introduce impurities due to process variables and trace elements in process feed stocks. These impurities can affect downstream conversion performance, compounds such as ash, char, tar and aromatics, lipophilic compounds that are known to accumulate into lipid bilayers affecting their functional properties [37]. Halogens and mono nitrogenous species such as hydrogen cyanide (HCN), ammonia (NH_3_), nitrogen oxide (NOx) and other known enzyme inhibiting gases such as acetylene (C_2_H_2_), ethylene (C_2_H_4_), ethane (C_2_H_6_) and oxygen (O_2_) can be present [3, 5, 6, 38, 39]. Sulfur compounds in the gas such as hydrogen sulfide (H_2_S), carbonyl sulfide (COS), carbon disulfide (CS_2_) can in turn negatively affect catalyst based scrubbing systems and their atmospheric release is restricted by environmental regulations.

For many of the above compounds commercially available scrubbing systems exist, however microbial gas fermentation as the downstream process is a relatively new addition. Monitoring optimal scrubbing system performance, including peak loads, saturation and regen cycles is critical to effectively maintain a reactive microbial population. A complete understanding of upstream process variability effect on gas contaminants production, together with the effect that accumulating and reactive impurities have, could reduce treatment costs. However, assuming feed gas process stability, at macro and micro composition, is an unrealistic expectation and can cause production delays at scale [40].

## Gas contaminant process tolerance

A distinct advantage of the biological conversion route is that a biocatalyst is versatile, constantly renewing due to its growth rate and as a consequence also capable of adapting to its environment. The biocatalyst is therefore less susceptible to poisoning by sulfur, chlorine and tar contaminants than inorganic catalysts which in turn have a much longer residence time, and therefore exposure to, the aforementioned gas contaminants [41, 42]. However tolerance levels to certain compounds is low, C_2_H_2_, HCN and NO are considered particularly troublesome as they are known to inhibit enzymes responsible for initial harvesting of carbon and energy from syngas in acetogenic organisms [43].

Hydrogen cyanide can be formed in gasifiers fed with nitrogen containing materials, and output concentrations can be influenced by gasifier operation parameters [44, 45]. Enzyme specific tolerance has been reported where cyanide specifically interacted with Fe-hydrogenases but not with di-nuclear metal centers as found in NiFe or FeFe hydrogenases [39]. In another study it was found cyanide acts as a competitive inhibitor acting on the Ni-4Fe-5S center of carbon monoxide dehydrogenase (CODH) [46], a key enzyme of the WLP [39, 44–47].

Besides cyanide, nitric oxide can be cogenerated in gasifiers. NO is a radical gas and used within biological systems as a transcriptional regulator [48]. At high concentrations however this reactive gas interacts within the cell to form toxic nitrogen oxides that inhibit key enzymes and at high concentrations prevent microbial growth [49]. A report on the inhibition of hydrogenase activity within a syngas operating system found tolerance levels to 40 ppm without compromising productivity while 200 ppm levels resulted in complete enzyme inactivation [49]. Biological tolerance can be based on conversion of NO to less reactive compounds such as nitrate (NO_3_) nitrous oxide (N_2_O) or ammonia (NH_3_) [48].

**Fig. 1:**
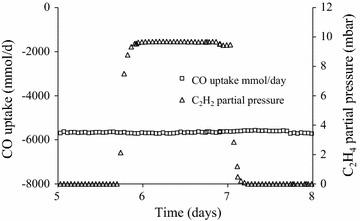
CO consumption profile of a continuously operating *C. autoethanogenum* gaseous fermentation undergoing addition of ethylene by sparging with ethylene containing Nitrogen. CO consumption remains stable around 5800 mmol CO/day

Acetylene dissolves well in aqueous solution, up to 47 mM at standard conditions and is a well-known inhibitor of metalloproteins due to reversible binding to the catalytic site [50]. Acetylene can reversibly inhibit hydrogenases limiting energy generation through H_2_ uptake [51, 52]. Due to the high reactivity with metalloenzymes tolerance levels are found to be low. Using 10% (v/v) C_2_H_2_ fed to *Rhodospirillum rubrum*, it was found that CO-linked hydrogenases had 50% reduced activity [52]. However it was found that only NiFe hydrogenases, not Fe hydrogenases, are inhibited by acetylene binding [51]. Using the rate of methanogenesis in marine sediments to study inhibitory compounds it was found acetylene irreversibly inhibits methane production while ethylene had a reversible inhibitory effect [53]. In the same study ethane was found to have no effect. Ethylene has also been described as a toxic compound to the gas fermentation process [38]. For commercialization of their gas fermentation process LanzaTech has performed extensive laboratory gas contaminant exposure tests on continuously grown *Clostridium autoethanogenum* cultures. Test results indicate ethylene appears to have limited to no effect on gas uptake rates in *C. autoethanogenum* cultures tested at up to a partial pressure of 10 mbar (Fig. 1).

**Fig. 2:**
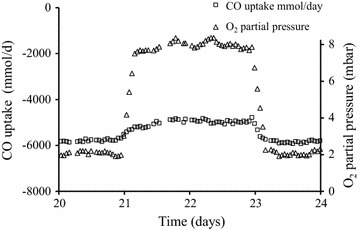
CO consumption profile of a continuously operating *C. autoethanogenum* gaseous fermentation undergoing varying levels of oxygen addition. At 2 mbar oxygen concentration CO uptake is stable at approximately 5900 mmol/day which, when oxygen is increased to 8 mbar, reaches a reversible equilibrium of CO uptake around 5000 mmol/day

For obligate anaerobic *Clostridium* species in industrial settings, oxygen and reactive oxygen species (ROS) are considered gas contaminants although some species are reported to withstand microoxic conditions [54–56]. In laboratory experiments on *C. autoethanogenum* under a partial pressure of up to 8 mbar oxygen an impact on CO utilization was measurable (Fig. 2). After reducing the oxygen concentration to 2 mbar the carbon monoxide uptake levels increased again indicating the tolerance level and reversible nature of the oxidative effect.

## Synthetic biology development

Synthetic biology and metabolic engineering approaches play an essential role in expanding acetogen product spectrum beyond the native products, such as ethanol, acetate and butanediol (BDO) to other fuels and commodity chemicals. These approaches had been applied to classic model microorganisms, such as *E. coli* and yeast which have been successfully engineered to produce non-native products at commercial scale [57–60]. On the other hand, acetogenic clostridia had long been considered challenging hosts for genetic modification. The slow development of reliable molecular biology tools is partly contributed by a strong native restriction-modification system, non-standard culturing conditions (toxic gas at pressure and obligate anaerobic), and slow doubling times. Since the successful demonstration of gas fermentation at pilot and pre-commercial scale as mentioned below, significant progress had been made in understanding acetogens at both the molecular and system biology levels [61–63]. Most notably, whole genome sequences, genome scale models, transcriptomic, proteomic studies and genetic tools have now been developed for these organisms. [18, 22, 26, 56, 61–71].

## DNA transfer

In order to genetically modify a microorganism, whether to delete a competing pathway or to introduce a new product pathway, it is imperative to have a reliable method to introduce foreign DNA into the cell. Electroporation and conjugation are the most frequently used methods for introducing foreign DNA into acetogens [26, 62, 72]. These strategies have been successfully demonstrated in *C. ljungdahlii*, *C. autoethangenum*, *C. aceticum*, *A. woodii* and *M. thermoacetica* [22, 62, 65, 73–76]. The highest transformation efficiency was reported to be around 1.7 × 10^4^ cfu/μg DNA for *C. ljungdahlii* in acetogens and the authors successfully introduced suicide vector with homology arms for chromosomal modification [62, 77]. Although electrocompetent cells preparation is elaborate, the method is donor cell independent, unlike conjugation. Further improvement of electroporation efficiency has been achieved through in vitro methylation or disruption of host’s restriction endonuclease, such as those examples in *C. acetobutylicum* [78], *C. pasteurianum* [79] and *C. cellulolyticum* [80], when the methylation/restriction patterns are identified either through restriction digestion pattern identification or PacBio sequencing [79, 81, 82].

In addition, conjugation is used broadly among *Clostridium* species, mainly because during conjugation DNA is transferred from donor to recipient cell as a single strand, not recognizable by the recipient’s restriction modification system. This method has been successfully used in *C. autoethanogenum* [26] and *A. woodii* [83]. In combination the two methods provide a robust basis platform for routine and advanced synthetic biology discovery.

## Genome modification

Homologous recombination utilizing host’s own recombination machinery is widely used for genome engineering. More specifically, a plasmid that carries homologous arms to the upstream and downstream areas of target gene(s), is introduced into the host. In order to select for a double crossover event (gene deletion), a positive selection (such as antibiotic resistance cassettes) or combination with a negative selection (such as *mazF* [84] or *pyrE* [85]) is used. Other variant methods that rely on homologous recombination also include Allele-Coupled Exchange (ACE) [86], Triple crossover [87] and scar-less, marker-less knockout or knock-in using two negative selection markers (*C. thermocellum*), detailed information has recently been reviewed [88]. In some instances, specific DNA sequences which can be recognized by site-specific recombinases, flanking the antibiotic resistance cassettes were introduced into the chromosome at the same time during the double crossover event. The antibiotic resistance cassettes can then be excised out of the chromosome by the site-specific recombinase and produce a marker-less mutant [77].

Other genetic modification tools utilizing RNA machinery, such as the group II intron gene inactivation [89] and CRISPR/Cas9 (Clustered Regularly Interspaced Short Palindromic Repeats/CRISPR-associated protein 9), a RNA-guided prokaryotic immune system which can cleave foreign DNA [90]. The group II intron method had been applied to different *Clostridium* species including acetogens such as *C. autoethanogenum* [26, 61], and others [91]. This method, based on RNA-mediated, retro-homing mechanism [89], provides a quick and easy gene inactivation tool without relying on host recombination machinery, thus bypassing the low occurrence of double crossover events, and resulted in greater success in genome editing in acetogenic *Clostridium*. However, the nature of group II intron mutagenesis is based on insertion of the group II DNA at the target gene, therefore, this method is flawed with the possibility of polar effects on downstream genes.

It was recently reported that the CRISPR/Cas9 system from *Streptococcus pyogenes* was successfully applied to acetogens, many other bacteria, and also yeast and Eukaryotes due to high and reliable efficiency, the simplicity in design and fast turnaround to generate scar-less mutants [90, 92–96]. Moreover, CRISPR/Cas9 system has been reported to target multiple genes at the same time (multiplex gene editing) [92], which allows for engineering bacterial strains with desired phenotypes in a one-step. This system has also reported to be able to edit bacterial strains at the single nucleotide level [97]. The CRISPR/Cas9 system has rapidly become the preferred method for genome editing in most organisms, facilitating rapid functional analysis and strain development for industrial applications.

## Genetic parts

In addition to chromosomal editing tools, genetic parts such as promoters, terminators ribosomal binding sites (RBS) [98, 99] are essential for both strain and pathway development. Unlike other model microorganisms for which commercial genetic parts and even software designing tools are available, acetogens’ part library is less well-developed, the majority of genetic parts such as the promoters are extracted from close *Clostridium* relatives or from its own genome. Recently inducible promoter systems had been successfully developed in *C. ljungdahlii* and *C. autoethanogenum*, respectively [25, 87]. It is critical to develop an organism specific validated library of genetic parts.

One limiting factor to carry out promoter screening in acetogens is the lack of fluorescent reporter protein that would allow signal to correlate with the amount of translation from a given quantity of mRNAs transcribed. So far, there has only been a flavin-based fluorescent protein derived from *Pseudomonas putida* that works under anaerobic conditions [100]. This has been used to characterize two endogeneous promoters of *C. cellulolyticum* [101]. However, it remains to be determined if this flavin-based fluorescence system will work in acetogens. Thus for most parts, promoters in acetogens are characterized using either the *gusA* or *catP* systems, encoding β-glucuronidase and chloramphenicol acetyltransferase, respectively [25, 87]. Characterizing promoter strength, based on the enzymatic activities, is however less straightforward and time consuming.

## Metabolic engineering in gas fermentation

Gas fermentation offers the benefit of not using heterologous feedstocks such as sugars that affect food supply chain. Metabolic engineering of acetogens in an industrial setting has been reviewed at length elsewhere [18]. The central metabolic pathway in acetogens begin with the reduction of CO/CO_2_ to acetyl-CoA through the WLP. Depending on the choice of strains and feedstocks used, various native products can be produced, including acetate, ethanol, 2,3-BDO, lactate, butyrate, etc. (Table 1 in [18] and reference therein). The metabolic profiles of acetate, ethanol and 2,3-BDO produced by various industrial strains have recently been summarized [19]. At LanzaTech a proprietary process has been developed that maximizes the conversion of CO to ethanol in *C. autoethanogenum* using steel mill off-gas. Furthermore, it has been demonstrated that deletion of the *budA* gene encoding for an enzyme catalyzing 2,3-BDO production resulted in an increase in ethanol selectivity and titer as a result of diminished production of 2,3-BDO [61, 102]. The ethanol pools currently produced from the demo plants around the world have been converted into the jet fuels by the catalytic process known as alcohol-to-jet, which involves dehydration to alkenes and oligomerization to the targeted C-length [103].

To enhance process viability, the conversion of gas to more valuable products than ethanol have to be developed. There have been several reported successes in expressing heterologous pathways to produce acetone, butanol, butyrate, and isopropanol, in acetogens [22, 25]. Recent publication by the White Dog Lab even employed a co-feeding strategy, producing a mix of acetone, isopropanol, ethanol, at 12.5 g/L in *C. ljungdahlii* with a combination of CO and sugar [104]. In addition to these products, LanzaTech has also developed and owns several patent families exemplifying the synthesis of higher value products such as 3-hydroxypropionic acid, methyl ethyl ketone, and mevalonate, by expressing corresponding biosynthetic pathway genes from photosynthetic bacteria *Chloroflexus aurantiacus, Klebsiella, E. coli* and even plant [105–108]. In most instances, the productions were demonstrated using a plasmid platform under the control of native promoter systems.

## Pathway and strain optimization

In order to scale up production, pathway gene expression needs to be optimized to minimize metabolic bottlenecks and un-wanted side products [109–111]. Even though the number of publications on this topic in the field of gas fermentation is limited, many of the approaches developed through the metabolic engineering of *E. coli* and yeast are applicable to the gas fermentation organisms. In general, the strategy involves multilayers of analysis and debugging, both at the biosynthetic pathway level as well as the overall metabolic flux level of the host cells [112, 113]. Due to the inherent complexity of a biological system, however, debugging bottlenecks one gene at a time is tedious and time consuming. Thus, it is more efficient to manipulate the gene expressions systematically, refactoring the biosynthetic pathway via modular design, combinatorial analysis and high-throughput screening, to identify the best combination of genes and promoters, and other transcriptional elements such as ribosomal binding sites (RBS), and terminators. [109, 114, 115]. Additionally, routine targeted proteomics and metabolomics can be performed to rapidly assess gene expressions and key metabolites accumulation [116–119]. With the technologies developed in the field of synthetic biology for the past 10 years, including computer-aided pathway design algorithms [120–122], DNA assembly and sequencing [123–125], it is now routine to screen a large combinatorial libraries. When combined with rational design and effective screening methodologies, the combinatorial library facilitates the search for ideal pathway combinations for highly productive strains [126, 127].

## Use of omics based technology to monitor bioprocess performance

Nextgen sequencing has become a powerful tool in process optimization. Routine sequence analysis at genomic and transcriptomic levels are carried out to determine gene expression and mutation rate, which directly relate to process productivity and stability at molecular level. One recent study linked the genomic and metabolic analysis of various acetogens to confirm the involvement of the acetaldehyde oxidoreductase (AOR) in ethanol production and NADPH-dependent alcohol dehydrogenase (ADH) in the hydration of acetone to isopropanol in acetogens [19]. Moreover, *C. autoethanogenum* has been the subject of a multi-omics investigation to compare energy metabolism between autotrophic and heterotrophic growth [61]. The study highlighted the interplay of hydrogenases and the electron-bifurcating Nfn complex in ethanol formation during the autotrophic growth. The study also concluded that the overall energy yield does not change during the autotrophic or heterotrophic growth. The vast data provided by omics analysis from production plants, can be used to further improve pathway and strain design.

Metabolic flux analysis is often used in conjunction to the omics analyses to debug bottlenecks through the metabolic flux of interest [128]. A metabolic flux analysis on the syngas species, *Clostridium tyrobutyricum*, correlated increase in NADH with increase in butanol production [129, 130]. Moreover, genome-scale metabolic flux balance analysis has been used to construct spatiotemporal metabolic models for *Clostridium ljungdahlii* [131]. When combined with the Optknock computation, the models could predict new gene knockout targets relevant to the overproduction of ethanol, lactate and 2,3-BDO in a bubble column reactor [132].

## Scale-up

As described above the research output in the gas fermentation field and the synthetic biology capabilities on its subject microorganisms have been rapidly expanding. However, in 2016 two of the three companies that own and operate scaled up gas fermentation facilities suspended operations. This immediately raises the question whether gas fermentation is scalable. Below we briefly summarize what is known about these three companies and for the first time present gas fermentation production data from a LanzaTech demonstration facility located within a steel mill plant in China.

Three companies, Coskata, INEOS Bio, and LanzaTech have operated pilot and demonstration plants for extended periods of time. Coskata’s technology reformed methane into syngas with a H_2_:CO ratio of between 2:1 and 3:1, followed by fermentation of this syngas to ethanol. This approach seeks to take advantage of the current low price of natural gas in geographies such as the US. While Coskata announced that it was to cease operation in 2015, the technology developed in this company now forms the basis of a new company: Synata Bio [133].

INEOS recently announced it is selling the INEOS Bio facility in Vero Beach, FL, USA [134]. This name-plate 8 million gallon per year (Mgy) semi-commercial facility was built as a joint venture with New Planet Energy Holdings, LLC. Commissioned in 2012, the facility used lignocellulosic biomass and MSW for generating syngas and coproduced 6 MW of electrical power. In July 2013 the company announced successful production of ethanol at its facility [135]. In September 2014 operational changes were imposed to optimize the technology and de-bottleneck the plant to achieve full production capacity [40].

## LanzaTech

**Fig. 3:**
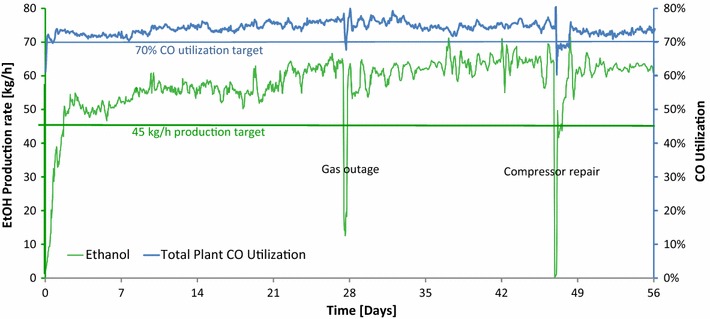
Ethanol production and carbon monoxide utilization profiles over an 8 week period. Data collected at the Beijing Shougang LanzaTech New Energy Science & Technology Co., Ltd, a 0.1 Mgy ethanol capacity demonstration facility

LanzaTech was founded in 2005 and after extensive piloting at a modest capacity steel mill plant in New Zealand, it partnered with 2 larger Chinese steel mills to build gas fermentation demonstration facilities. The first Demonstration unit was located at one of BaoSteel’s mills near Shanghai (operational since 2012) and the second at a Shougang steel mill near Beijing (operational since 2013), both facilities have a 100,000 gpy pre-commercial capacity. Typical production results from the second facility (Fig. 3) are shown below. To our knowledge this is the first time continuous, long term gas fermentation production data has been published from a demonstration facility. It is important to note this facility is running directly off steel mill produced off-gas and operational set-ups are a reality of scaled up operations. The gas fermentation process has proven robust to a wide variety of process upsets such as: macro gas concentration fluctuations, presence of gas contaminants, intermittent gas supply and equipment failure which can be replaced during the continuous fermentation.

The Shougang facility earned the Roundtable on Sustainable Biomaterials (RSB) certification for sustainability [136]. The RSB is the most robust and credible global sustainability standard and certification system for biofuels and biomaterials production. Here we present production and gas utilization data from a typical run from the RSB certified plant. The resulting ethanol from the LanzaTech Demo facilities has been turned into jet fuel ready for a test flight scheduled for 2017 [137].

In 2015, both China Steel Corporation of Taiwan and ArcelorMittal of Luxembourg approved commercial projects with LanzaTech. The former will be a 17 Mgy facility with the intention to scale up to 34 Mgy [138]. The latter 21 Mgy facility will be built at ArcelorMittal’s flagship steel plant in Ghent, Belgium with intention to construct further plants across ArcelorMittal’s operations [139]. If scaled up to its full potential at steel mills in Europe alone, the technology could enable the production of around 104 Mgy with the potential to displace 1.6 million barrels of fossil fuel-derived gasoline on a BTU basis.

## Summary and outlook

Gas fermentation is rapidly becoming an established platform for the conversion of (waste) gas to valuable liquid chemicals. Clear advantages are process stability and tolerance to inhibitory compounds and therefore flexibility in gas feedstock sourcing. Process upsets, either upstream or downstream can occur with limited warning at scaled up operations. Resilience of the microbial culture to upsets can be enhanced by engineering design to limit their impact. The production of ethanol has been proven robust at scaled up operations, the next stage is now set for expanding the product portfolio utilizing advanced synthetic biology technologies developed for gas fermenting microorganisms. This allows for a profitable carbon recycling operation, producing sustainable chemicals independent of carbon credits, to further limit GHG emission. With an industrially robust strain, efficient genetic toolbox, advanced synthetic biology capabilities, and scalable reactor design, the field of gas fermentation remains on course to reduce global carbon emissions.
